# MULTISECTOR PLANS FOR AGING: RESEARCH AND STATE GOVERNMENT PARTNERSHIPS TO TRANSFORM AGING SERVICES

**DOI:** 10.1093/geroni/igad104.1902

**Published:** 2023-12-21

**Authors:** Carrie Graham

**Affiliations:** Center for Health Care Strategies, Hamilton, New Jersey, United States

## Abstract

As the US population becomes older and more ethnically diverse, state leaders are increasingly seeing the need to conduct high-level, cross-sector planning to meet the needs of their aging populations and promote equity. A state Multisector Plan for Aging (MPA)-- also called a master plan or strategic plan-- creates a valuable roadmap that can help states transform the infrastructure and coordination of services for all people who are aging in the state. Developing an MPA requires a state-led process that brings various state agencies (Aging, Medicaid, Public Health, Social Services, Housing, Transportation, etc.) together with stakeholders and researchers to outline a clear framework for addressing the needs of older adults, people with disabilities, and caregivers, for 10 years or more. The growing movement of states developing MPAs offers a unique opportunity for aging researchers to partner with state policymakers and stakeholders to ensure that the state’s MPA is grounded in evidence and that implementation progress is evaluated using appropriate data and benchmarks. This paper describes the experience of several states that are developing MPAs and how aging researchers in the state have successfully partnered with policymakers, using their research to help the state garner buy in for developing an MPA, elevate the most pressing issues, project population characteristics and needs in the future to inform the development process. Individual researchers, universities and research subcommittees working with states on MPA development can also partner to mine available state data, identify benchmarks, and create data dashboards to guide implementation and monitoring progress.

